# Nurses’ perspectives on the adoption of new smart technologies for patient care: focus group interviews

**DOI:** 10.1186/s12913-025-12578-z

**Published:** 2025-03-18

**Authors:** Hyein Choi, Sunghee H. Tak, Young Ae Song, Jiyeon Park

**Affiliations:** 1 College of Nursing, Seoul National University, 103 Daehak-ro, Jongno-gu, Seoul, 03080 South Korea; 2 Research Institute of Nursing Science, College of Nursing, Seoul National University, 103 Daehak-ro, Jongno-gu, Seoul, 110- 799 Republic of Korea; 3 Department of Nursing, Seoul National University Bundang Hospital, Seongnam-si, Gyeonggi- do South Korea

**Keywords:** Technology adoption, information technology, Smart health, Clinical nursing, Patient care

## Abstract

**Background:**

The adoption of smart technologies for patient care is greatly increasing. IoT-based smart mattresses offer features such as automatic body repositioning for pressure ulcer prevention, weight measurement, vital sign tracking, and rapid response to emergencies. This study explores nurses’ perspectives on the adoption of smart technologies, with a particular focus on smart mattresses in hospitals.

**Methods:**

Focus group interviews were conducted with 17 nurses from intensive care parts, general wards, and COVID-19 designated wards in a tertiary university hospital in South Korea. Data were collected through online meetings and analyzed using thematic analysis.

**Results:**

Thematic analysis revealed six major themes and 14 subthemes under three categories. Patient-related perspectives included themes of ‘difficulties in meeting patients’ care needs’ and ‘identified areas for technology adoption based on patient care experiences.’ Nursing-related perspectives encompassed ‘potentials of smart technology for nursing care’ and ‘increased time and workload due to new device usage.’ Technology-related perspectives included ‘previous experience with technology devices’ and ‘expectations for in-development devices.’

**Conclusions:**

Nurses generally expressed receptiveness to smart technologies including smart mattresses, recognizing their potential to enhance patient care and nursing efficiency. Perspectives varied by department, highlighting the need for tailored solutions. Prior experiences, both positive and negative, offered important insights for designing devices that are more compatible with clinical practice. Incorporating nurses’ feedback and addressing educational needs are critical for successful adoption.

**Supplementary Information:**

The online version contains supplementary material available at 10.1186/s12913-025-12578-z.

## Background

The rapid development of smart technologies is transforming bedside patient care [1]. These technologies refer to a wide range of innovations that integrate advanced digital systems and Internet of Things (IoT) capabilities to improve patient care and clinical efficiency [2]. The technologies include smart beds, automated IV(Intravenous) pumps and automated monitors, all designed to improve clinical efficiency and patient safety [3]. Many of these technologies assist nurses with routine tasks while reducing human errors, particularly in situations where overloaded nurses manage long shifts with many patients [4]. In general, new technology and equipment innovation is expected to allow the delivery of healthcare services to be efficient and positively impact patient care [5].

Among these innovations, smart mattresses with internet of things (IoT), have emerged as a key solution for bedside care because they can be designed as the platform or hub of all the technologies for patient care bedside [6]. Smart mattresses offer advanced features such as automatic body repositioning to prevent pressure ulcers, real-time weight monitoring, and vital sign tracking. These functions not only enhance patient safety but also help alleviate the physical burden on nurses, who frequently perform tasks such as patient lifting and repositioning [7]. In general, clinical wards in hospitals are likely to use either the beds only with angle adjustments using remote control or in-bed scales for patients who cannot move [8]. In intensive care units, electric beds with weight measure function are often used. Recently, the demand for high quality medical beds and mattresses with various functions is increasing [9]. In particular, as the society transitions into an aging population, the demand for advanced medical technologies, such as smart mattresses, continues to grow to address the increasing healthcare needs of older adults [10].

While in the hospital, a patient’s bed is a space where he or she spends the most time and also the place in which bedside care is performed by nurses. Nurses face significant challenges related to repetitive tasks in bedside care, including vital sign measurement, position change and body weight measurement [11]. The vital signs and weight of severely ill patients are very important clinical indicators to understand changes in the patient’s condition [12]. However, these tasks, while essential for preventing pressure ulcers and monitoring patient conditions, contribute to musculoskeletal disorders (MSDs)—a prevalent issue among nurses globally [13, 14]. Studies have shown that approximately 74% of Korean nurses experience MSDs, particularly in the back, waist, and shoulders, due to physical strain [15, 16]. An innovative approach with new smart devices and equipment that nurses can use is essential in order to reduce the physical burden of the nurse such as patient lifting due to weight measurement and posture change [17, 18].

In addition to the physical burden, patient safety remains a major concerns [19]. Bedsides are common sites for patient falls, which can lead to severe injuries [20]. Furthermore, nurses caring for patients with infectious diseases face increased risks of exposure, as bedside care often involves frequent physical contact [21]. During recent outbreaks, such as MERS and COVID-19, minimizing patient contact while ensuring high-quality care has become a top priority [22]. New technologies, such as IoT-based smart mattresses, may address these challenges by automating routine processes, improving patient monitoring, and reducing the risk of infection transmission [23].

Given that a patient’s bed is central to bedside care, adopting technologies that optimize this space represents a logical step toward improving clinical efficiency and patient outcomes [8]. As Roger’s Diffusion of Innovations highlights, factors such as perceived advantages and compatibility are critical to the successful adoption of these technologies [24]. In this context, IoT-based smart mattresses offer practical benefits by automating routine care processes, reducing the physical workload of nurses, and enhancing patient safety. Therefore, the purpose of this study was to explore nurses’ perspectives on the adoption smart healthcare technologies in hospitals, with a particular focus on IoT-based smart mattresses as a representative example of these innovations. To achieve this, the study aimed to answer the following research questions:

1. What are nurses’ experiences and perceptions regarding the adoption of IoT-based smart mattresses for bedside patient care?
2. What challenges do nurses face when using smart healthcare technologies, including IoT-based devices, in clinical settings?
3. What are nurses’ expectations for improving the usability and effectiveness of IoT-based smart mattresses and other smart healthcare technologies?

## Methods

### Study design

This study adopted a qualitative approach grounded in the interpretivist paradigm to explore nurses’ experiences and perceptions regarding smart mattresses through focus group interviews(FGI). FGIs were conducted among nurses who worked at a tertiary hospital. Study participants participated in online meetings due to the restriction policies of hospital visits related to COVID-19. Zoom was selected based on its convenience, ease of use, smooth interaction, and security features [25, 26]. Previous research has demonstrated that online FGIs produce data quality comparable to in-person sessions, ensuring reliable data collection while facilitating open discussions on sensitive topics [27, 28]. This study adhered to the Standards for Reporting Qualitative Research (SRQR) guidelines to ensure transparency and rigor in the reporting of qualitative methods and findings [29].

### Study participants

Study participants were nurses recruited from a tertiary hospital. The eligibility criteria included previous experience of changing patients’ posture at intervals of 4 h, measuring body weight of patients on beds every day, and monitoring vital sign at intervals of 4 h within one year. Nurses who had clinical career less than 1 year were excluded.

A total of 17 nurses who met the criteria participated in the study. Of the study participants, three are men and fourteen are women. All participants have a bachelor degree in nursing and consisted of intensive care unit nurses(*n* = 5), ward nurses(*n* = 8), nationally-designated inpatient ward for infection nurses(*n* = 4). Regarding participants’ clinical experiences, the years of their clinical practice ranged from 20 months to 119 months (see Table 1). A smart bed with features such as angle adjustments and in-bed weight measurement had already been introduced in the ICUs of the participating hospitals, and 4 out of 17 participants reported prior experience with this bed; however, it does not include the automatic lateral repositioning function featured in the IoT-based smart mattress examined in this study.

**Table 1:** Selected nursing care performed by study participants (*N* = 17)

Nursing care	Number of performance	Number of patients
	Mean(SD)	Mean (SD)
Changing a patient’s body position (per week)	31.8 (28.6)	10.8 (8.2)
Checking vital signs (per day)	12.0 (6.7)	4.7 (4.5)
Measuring a bedridden patient’s body weight (per day)	2.4 (1.9)	2.4 (1.9)

The patient care tasks performed by the participants included body position changes, vital sign checks and body weight measurements. On average, they conducted postural changes for 10.76 persons (SD 8.19) in the past week, totaling 31.76 times (SD 28.59). They monitored vital signs for an average of 4.68 persons per day (SD 4.50) over the past month, totaling 11.94 times per day (SD 6.67). Body weight measurements on the bed were taken for an average of 2.38 persons per day (SD 1.92), totaling 2.38 times per day (SD 6.67) in the past month (see Table 1).

### Data collection

Upon approval of the Institutional Review Board, recruitment flyers were distributed to potential participants using the hospital’s electronic notification system. Participants voluntarily contacted the researcher via e-mail or mobile phone. Study participants were informed about the purpose of the research and informed consent was obtained prior to participation.

FGIs were conducted with 17 participants across 4 groups organized by work departments; two intensive care wards (intensive care units and intensive care rooms), one nationally-designated inpatient ward, and one orthopedic ward. Nationally-designated inpatient ward was specialized infectious disease management facility with compartment designed to prevent pathogen transmissions, particularly COVID19. Previous studies suggest that no more than 6 people per group and three or four groups are adequate for focus group interview depending on the complexity of the topic and the degree of diversity of topics [30]. In this study, each group consisted of three to five participants.

After completing questionnaires related to participants’ general characteristic, a semi-structured interview was conducted using an interview guide (refer to a supplementary file). The focus group interviews (FGIs) were conducted by a researcher with three years of nursing experience who had no prior relationship or conflicts of interest with the participants. The researcher completed training in qualitative research methods, including focus group facilitation, prior to the study. The interview lasted 60 to 90 min, and all interviews were recorded via the Zoom with participants’ consent. The recordings were transcribed verbatim for analysis.

### Data analysis

The total word count for the text data varied across groups; Group 1: containing 6,283 words, Group 2: 4,885 words, Group 3: 6,136 words, and Group 4: 5,157 words. Qualitative data from interviews were analyzed using the thematic analysis. Thematic analysis is useful for focusing on identifying themes within conversations [31]. Data were analyzed by two researchers according to the six steps of thematic analysis [28]. First, the recorded data were immediately transcribed after the interview. Then, two researchers repeatedly read transcription records, derived meaningful sentences, and coded them, and had a consensus process. Categories and subcategories were derived from the meaningful statements. The entire data was repeatedly reviewed, reaffirming whether the statements were consistent with the categories and subcategories. Each category is named and defined. Finally, a report was written based on the results of the analysis.

### Ethical considerations

This study was approved by the Institutional Review Board. Focus group interviews were conducted in January 2021. Informed written consent was obtained from the participants. Personal information remained anonymous and participants had the freedom to withdraw from the study at any time. The collected data were maintained in a non-identifiable format and securely stored on the computer with a double-locked system that included both computer and document safeguards. All data will be retained for three years following the completion of the study, in accordance with institutional and ethical guidelines, after which they will be permanently deleted to ensure participant confidentiality.

## Results

From the data analysis, six major themes with 14 subthemes emerged, categorized into three areas: Patient-related perspectives on smart technology focusing on smart mattresses, nursing-related perspectives on smart technology focusing on smart mattresses, and Technology-related perspectives: General experiences and expectations. In relation to patients, the major themes encompassed ‘difficulties in meeting patients’ care needs’ and ‘identified areas for technology adoption based on patient care experiences.’ Concerning nursing, the major themes included ‘potentials of smart technology for nursing care’ and ‘increased time and workload due to new device usage.’ Finally, the major themes associated with technology were ‘previous experience with technology devices’ and ‘expectations for in-development devices.’ A detailed representation of the major themes and subthemes can be found in Table 2.

**Table 2:** Qualitative analysis and summary of nurses’ perception on the adoption of new smart devices

Categories	Major theme	Subtheme
Patient-related perspectives on smart technology focusing on smart mattresses	• Difficulties in meeting patients’ care needs	- Recognizing patients’ diverse care needs which need to be addressed simultaneously and urgently - Perceiving the need for smart technology in patient care
	• Identified areas for technology adoption based on patient care experiences	- Changing patients’ positions and preventing pressure ulcer without disturbing their sleep - Involving patients’ needs for comfort and safety
Nursing-related perspectives on smart technology focusing on smart mattresses	• Potentials of smart technology for nursing care	Optimizing work efficiency - improving the quality of care
	• Increased time and workload due to new device usage	- Responding to malfunction and incidents - Learning numerous new functions - Backing up which is often needed (e.g., when using IoT devices) - Addressing numerous questions from patients and caregivers
Technology-related perspectives: General experiences and expectations	• Previous experience with technology devices	- Experiencing frequent false alarms - Enhancing work efficiency and convenience due to the integration of smart technology
	• Expectations for in-development devices	- Hoping for enhanced accuracy and precision - Anticipating consistency in measurements

### Patient-related perspectives on smart technology focusing on smart mattresses

#### Difficulties in meeting patients’ care needs

Nurses expressed shared concerns about the challenges of meeting the patients’ care needs. Specifically, they highlighted the difficulties in recognizing and promptly addressing various patient requirements. These challenges included the urgency required in responding to such needs and the critical role of smart technology in facilitating their timely identification.

##### Recognizing patients’ diverse care needs which need to be addressed simultaneously and urgently

Nurses discussed the challenges of recognizing and addressing a variety of needs, some of which may feel urgent to patients but appear minor or non-emergent from the nurses’ perspectives. For example, one nurse shared, “In the intensive care unit, I noticed a significant issue. From the patient’s standpoint, enduring a wet diaper without immediate attention can be extremely uncomfortable. The sensation on the skin is distressing, and wearing a diaper while fully alert is particularly challenging.” In practice, non-critical needs are often deprioritized in nursing care, potentially impacting to adverse consequences for patients’ overall well-being. One nurse reflected on this, “The primary focus tends to be solely on preventing pressure ulcers, to the extent that even patients who are sleeping well are woken up, disrupting their rest. I often wonder, ‘When can this patient get more than three hours of uninterrupted sleep?’ My perspective on providing quality patient care has evolved. I’ve come to realize that even if a patient has mild pressure ulcers, they may be in desperate need of rest.”

##### Perceiving the need for smart technology in patient care

The research participants recognized the necessity of utilizing smart technology to address the diverse and non-critical needs of patients, which are often challenging for nurses to manage promptly. One participant suggested, “I even thought about features that could automatically change a wet diaper. Alternatively, if a smart mattress were equipped with a sensor, it could trigger an alarm when a patient’s diaper becomes wet.” Another participant proposed, “I believe a smart device could help alleviate some of the back pain experienced by patients by supporting back flexion through the controlled application of air.” These insights underscore the growing demand for innovative technological solutions to better accommodate the multifaceted needs of patients.

#### Identified areas for technology adoption based on patient care experiences

During the interviews, the nurses identified areas where technology could be effectively applied based on their patient care experiences. Notably, they acknowledged technology’s potential to assist with patient repositioning in bed and prevent pressure ulcers, all while maintaining uninterrupted patient sleep. Additionally, they recognized opportunities for technology to improve both patient safety and comfort.

##### Changing patients’ positions and preventing pressure ulcer without disturbing their sleep

Nurses, particularly those working in intensive care units, often encounter situations where they must reposition patients to prevent pressure ulcers, sometimes at the cost of the patient’s sleep quality. The introduction of an automated position-changing function could significantly improve sleep quality by minimizing nurse interference. As one nurse stated, “If a smart mattress can automatically change the position of patients, I don’t think we’re doing anything wrong even if we don’t physically touch them at night. The presence of automated position support features could enhance patients’ sleep quality.”

##### Involving patients’ needs for comfort and safety

Nurses acknowledged that smart technology could contribute to improving patient safety indicators, particularly in addressing concerns such as falls and infections. One nurse shared, “Falls can still occur despite our best efforts, so having a smart device as a warning guide would be beneficial.” Another nurse stated, “If the bed is equipped with X-ray plates, I believe the frequency of plate cleaning could be reduced when caring for infected patients.” These insights highlight the potential of smart technology to enhance patient safety and reduce the incidence of falls and infections.

### Nursing-related perspectives on smart technology focusing on smart mattresses

#### Potentials of smart technology for nursing care

Nurses highlighted the potential of smart technology to enhance their work efficiency and improve the quality of care.

##### Optimizing work efficiency

Given the characteristics of nursing, where healthcare professionals interact with numerous patients, there is strong interest in technologies that can minimize unnecessary tasks and optimize the use of their valuable nursing time. In particular, the integration of Electronic Medical Records (EMR) with measurement devices was seen as a way to maximize work efficiency. One nurse emphasized, “Automatically interlinking vital signs data increases work efficiency and offers convenience.” Furthermore, nurses working in general wards without beds equipped with weight-measurement functions stressed the need for such features to improve efficiency. One nurse remarked, “There is considerable interest in when the beds with weight-measurement functions will be introduced, as they are consistently needed in our ward.” Another nurse added, “Considering the significant energy consumption, I hope beds with weight measurement functions can be introduced to enhance work efficiency.”

##### Improving the quality of care

Nurses expressed optimism that the adoption of smart technology could alleviate the burden of nursing work and potentially enhance the quality of care, leading to an positive outcomes for patients. One nurse expressed, “I believe that these devices can improve the quality of nursing care by allowing us to focus on other aspects while reducing the physical strain on our backs.” Another nurse emphasized the potential benefits, stating, “Even if technology provides only modest assistance, it can still contribute to delivering higher-quality care.” These perspectives highlighted the potential synergy between smart technology and nursing care quality.

#### Increased time and workload due to new device usage

Nurses, who frequently interact with various medical devices and continually adapt to new technologies tailored to their workplace’s characteristics, acknowledged that the introduction of smart technology presents its own challenges alongside its advantages.

##### Responding to malfunction and incidents

Managing safety incidents caused by device malfunctions and inexperience with new smart devices emerged as the primary concern among nurses, in particular, expressed heightened apprehension about the risks of device malfunctions due to the potential for fatal outcomes in such high-stakes environments. An ICU nurses stated, “We are deeply concerned about the possibility of fatal consequences in the ICU if a machine malfunction occurs.” Meanwhile, nurses in general wards concerns about the challenges posed by the diverse range of smart devices from different manufacturers, which complicates maintaining strict supervision. One nurse in a general ward shared, “In general wards, some patients are good at using devices, but those who are not may struggle. This disparity in user proficiency increases the risk of device breakdowns or accidents when patients attempt to operate them.”

##### Learning numerous new functions

Introducing new smart devices also imposes additional responsibilities on nurses, with some expressing concerns about the burdens associated with adapting to new functionalities. One nurse remarked, “While the Electronic Medical Records (EMR) system is beneficial, I believe it adds to our workload due to its extensive functionality.” Another nurse elaborated, stating, “The abundance of features within smart devices create more tasks for us to manage.” These perspectives highlight that challenges that can arise when integrating new technologies into nursing practice.

##### Backing up which is often needed (e.g., when using IoT devices)

Another perspective raised was the potential increase in workload if the device’s accuracy is difficult to trust, requiring nurses to verify and ensure data correctness, essentially doubling their tasks. This viewpoint reflects a negative perception of the interlocking function, as one nurse explained, “Even if the machine takes a measurement, it becomes a double task when nurses need to independently validate and filter the data through monitoring.”

##### Addressing numerous questions from patients and caregivers

Nurses in general wards, where clear communication with patients and caregivers is essential, expressed concerns about the increasing workload associated with an addressing numerous inquiries prompted by the introduction of smart technology. One nurse shared their experience, stating, “The alarms keep ringing, and caregivers constantly ask about the purpose of each alarm and its associated function. To be honest, responding to these inquiries can be quite burdensome.” Another nurse added, “In the general ward, if all patients use smart mattresses, I think they’ll be ringing all the time…”.

### Technology-related perspectives: general experiences and expectations

#### Previous experience with technology devices

Participants mentioned negative and positive experiences with smart technologies or devices that they had used in nursing work before the interview.

##### Experiencing frequent false alarms

Many nurses expressed negative perceptions about false alarms caused by accuracy issues with medical devices. One nurse noted, “The respiratory rate is often measured incorrectly, and the apnea alarm rings so frequently that I often find it necessary to turn it off.” Another added, “There have been numerous instances where the apnea alarm sounds falsely, even on the current monitors.”

##### Enhancing work efficiency and convenience due to the integration of smart technology

Despite the negative experiences associated with the introduction of smart technology, participants acknowledged both the convenience and ability to enhance work. One nurse remarked, “I haven’t experienced delays in my work because blood pressure and ECG are automatically measured and integrated with the EMR.” Notably, technology was recognized as a valuable complement to human actions, enhancing the accuracy of patient identification and significantly enhancing patient safety. A nurse elaborated, “It’s reassuring to have devices scan the patient label first to ensure the correct medication is administered to the right patient, reducing the risk of medication errors.”

#### Expectations for in-development devices

Participants expressed expectations for devices currently under development, viewing them as potential solutions to the limitations of existing devices on prior usage experience. In particular, issues related to the accuracy of existing measuring devices led to widespread anticipation for enhanced accuracy and measurement consistency in new devices, especially for weight- measuring systems.

##### Hoping for enhanced accuracy and precision

There was optimism that advancements in the accuracy of vital signs measurement could help alleviate the nursing workload. As one nurse noted, “Accurate measurements of apnea, cardiac arrest, sleep patterns, and vital signs can significantly reduce the workload.” Another nurse emphasized the need for improved accuracy, stating, “It’s challenging to respond to all of the questions from caregivers about vital sign alarms, so I hope the measurements are accurate to address this issue”.

##### Anticipating consistency in measurements

ICU Nurses with experience using beds equipped with weight-measuring capabilities expressed a desire for greater consistency in weight measurements. One nurse explained, “When discussing weight-related matters, we sometimes find ourselves trying to adjust the values to fit into our input/output (I/O) records.” Another nurse highlighted the challenges specific to the ICU, stating, “The most significant difficulty in the ICU is measuring weight, and the variability in patient’s weight, even under the same conditions, is quite pronounced.”

## Discussion

This study explored nurses’ perceptions regarding the adoption of smart technology focusing on IoT-based auto-rotating technology. The findings of this study revealed nurses’ perspectives regarding the adoption of new smart devices for patient care. The identified themes and subthemes indicate that nurses perceive new smart technology as a means to address patient care needs, while also recognizing both positive and negative aspects in the actual use of the technology.

Nurses, the participants in this study, demonstrated a keen interest in addressing the challenges that arise in patient care and the potential of new technologies to partially mitigate these challenges. The nurses were acutely aware of the difficulties in meeting the diverse needs of patients, recognizing disparities between the patients’ perceived priorities and their own. This finding aligns with previous studies highlighting discrepancies in the perception of care needs between nurses and patients [32]. It reflects a mindset aimed at efficiently managing the individual differences inherent in care requirements [33]. Therefore, the introduction of new smart technology was regarded as a means to alleviate difficulties arising from such discrepancies.

However, the study participants also expressed ambivalent feelings regarding new smart devices. In this study, nurses anticipated that the application of smart mattresses would alleviate the nursing workload, but they also expressed concerns about the potential increase in nursing tasks due to the introduction of new technologies. This ambivalence toward technology has been a longstanding subject of discussion [34]. A similar pattern was observed in previous research related to the use of humanoid robots in the medical field, where researchers noted ambivalent emotions, such as ‘safe and unsafe’ and ‘reliable yet unreliable,’ associated with the technology [35].

The findings highlighted the influence of prior experiences on the adoption of new devices. Previous studies reported that experiences influence directly or indirectly to their acceptance of technology [36, 37]. In this study, when sharing their experiences about existing technologies, many nurses have positive opinions on the interwork functions of smart devices with EMR. They expected that interworking technology would reduce the burden on nurses by replacing small tasks that nurses have to do by themselves. The accumulation of positive experiences with digital technology is crucial, as positive experiences in the realm of medical technology can significantly influence attitudes and motivations to embrace and utilize technology in the workplace [38]. Nevertheless, this study also affirmed that negative experiences with technology can be valuable, as nurses can offer insights for devising and improving new technology. In this study, nurses’ negative experiences led to suggestions for additional features and new ideas for smart mattresses. Additionally, the educational needs of patients and caregivers were identified to enhance their digital literacy and reduce increased inquiries caused by the use of smart devices.

Findings revealed that nurses’ perceptions of smart mattresses were influenced by their broader experiences with healthcare technologies, making it difficult to draw a clear distinction between smart mattresses and smart technologies overall. While the study focused on smart mattresses, participants frequently referenced functionalities from existing systems, such as interworking technology and monitoring devices, when discussing potential benefits or improvements. This indicates that their attitudes toward adopting smart mattresses were shaped by cumulative experiences with related technologies, reflecting the interconnected nature of technology adoption in healthcare. These findings align with prior research highlighting that both positive and negative experiences influence attitudes toward new technologies.

Since nurses are in close proximity to patients and spend the most time with them, in this study, they expressed the desire to address a wide array of patient needs. While they had an ambivalent perception of smart devices, they generally exhibited a receptive attitude. However, it is important to introduce medical devices capable of catering to the diverse needs of different nursing work settings. A recent study reported the gap between intensive care units and wards in terms of the desired functions of technology [38]. This gap is stemmed from the difference in the technologies like the types of beds they use, monitoring devices, and in the number of patients a nurse had assigned. Nursing administrators and device manufacturers should carefully consider the healthcare context and prioritize new technology adoption by actively soliciting user feedback [39].

### Strengths and limitations

This study has several strengths. First, it addresses an emerging area of healthcare technology by focusing on nurses’ perceptions of IoT-based smart mattresses, providing valuable insights into their potential for improving patient care and nursing workflows. Second, the inclusion of nurses from diverse clinical settings, such as ICUs, general wards, and infection-control units, enriches the findings by capturing a range of perspectives and needs. Third, the use of rigorous qualitative methods, including focus group interviews and thematic analysis, ensures an in-depth exploration of participants’ experiences. Finally, the study offers actionable insights for technology developers and healthcare administrators, contributing to the practical implementation of smart devices in clinical settings.

This study has some limitations. First, participants’ responses were influenced by prior experiences with existing devices, potentially limiting the generalizability of findings to all nursing contexts. Second, the study was conducted in a single tertiary hospital, which may affect the transferability of results to other healthcare settings. Third, differences in clinical backgrounds and departmental needs among participants highlight the need for further studies with larger, more diverse groups. Fourth, This study was conducted in 2021 during the COVID-19 pandemic, a period of rapid digital transformation in healthcare. While this context provided unique insights, the findings may not fully reflect post-pandemic perceptions as technological norms and workflows continue to evolve. Finally, while rigorous thematic analysis was employed, the researchers’ background may have influenced interpretation, despite efforts to minimize bias.

## Conclusion

This study investigated nurses’ perceptions regarding smart technologies, focusing on smart mattresses with IoT-based auto-rotating features for patient care in hospitals. The findings highlighted perspectives across three areas: Patient-related perspectives on smart technology focusing on smart mattresses, nursing-related perspectives on smart technology focusing on smart mattresses, and Technology-related perspectives. Nurses generally held a receptive view of smart technologies including smart mattress, identifying their potential to address patient care needs and improve work efficiency. Variations in perception across departments underscored the need for tailored technological solutions. Future implementations should actively incorporate nurses’ feedback to optimize functionality and usability. Positive and negative experiences with prior technologies provided valuable insights for developing devices that better align with clinical practice. Future studies should focus on conducting clinical trials and addressing educational needs to ensure successful adoption and integration of smart devices in diverse healthcare settings.

## Supplementary Information

Supplementary Material 1.

## Data Availability

The datasets used and/or analyzed during the current study are available from the corresponding author on reasonable request.
