# Supplementary material for: Nurses’ perspectives on the adoption of new smart technologies for patient care: focus group interviews
Source: BMC Health Serv Res. 2025 Mar 18;25:391. doi: 10.1186/s12913-025-12578-z (PMC11917002; doi:10.1186/s12913-025-12578-z)
Supplement: Supplementary file 1 — Supplementary Material 1. [file 12913_2025_12578_MOESM1_ESM.docx]

**Interview Guide for Focus Groups**

[Opening Questions]

- If you have any experiences where technology has helped alleviate the burden of nursing duties, please share. Additionally, what tasks do you think technology could be utilized for to alleviate nursing burdens?
- When you hear the term 'Smart Mattress,' what functionalities do you think it might include?

[Key Research Questions]

Please freely express your opinions on the development and potential usage of a Smart Mattress with the following functionalities in clinical nursing: (1) measurement of patient biometric data (weight, respiration, heart rate, sleep state, etc.) (2) automatic rotation for position changes (every 2 hours, shifting 30 degrees, lateral and angular changes) (3) standard interface for integration with hospital medical information systems, equipped with IoT platform.

- What are your thoughts on the practicality of the Smart Mattress? What aspects are you looking forward to?
- Which functionalities do you believe should be added or emphasized?
- What additional functionalities do you think would be beneficial but not necessarily essential for the Smart Mattress? Why?
- What do you think about the introduction of the Smart Mattress into nursing practice?
- What functionalities do you think are necessary to increase the efficiency of nursing processes (assessment, care provision, nursing evaluation, etc.) with the Smart Mattress?
- How do you think the Smart Mattress will affect in terms of both patient health and safety and nursing duties?
- Which functionalities do you think will have a significant impact on nursing burdens (e.g., physical workload)?
- Please also discuss the impact of the Smart Mattress in situations like COVID-19.
- Are there any concerns if the Smart Mattress is introduced and utilized for patient care in hospital wards?
- What potential problems do you think may arise in nursing duties due to the introduction of the Smart Mattress? If there are anticipated issues, what solutions do you propose?
- What considerations should be taken into account regarding design, functionality, and technical support to enhance the utility of the Smart Mattress?

[Closing Question]

If you have any additional opinions or thoughts you would like to add based on the discussion, please feel free to share.
